# Supplementary figures and images for: Genome-Wide Characterization of the MLO Gene Family in Cannabis sativa Reveals Two Genes as Strong Candidates for Powdery Mildew Susceptibility
Source: Front Plant Sci. 2021 Sep 13;12:729261. doi: 10.3389/fpls.2021.729261 (PMC8475652; doi:10.3389/fpls.2021.729261)

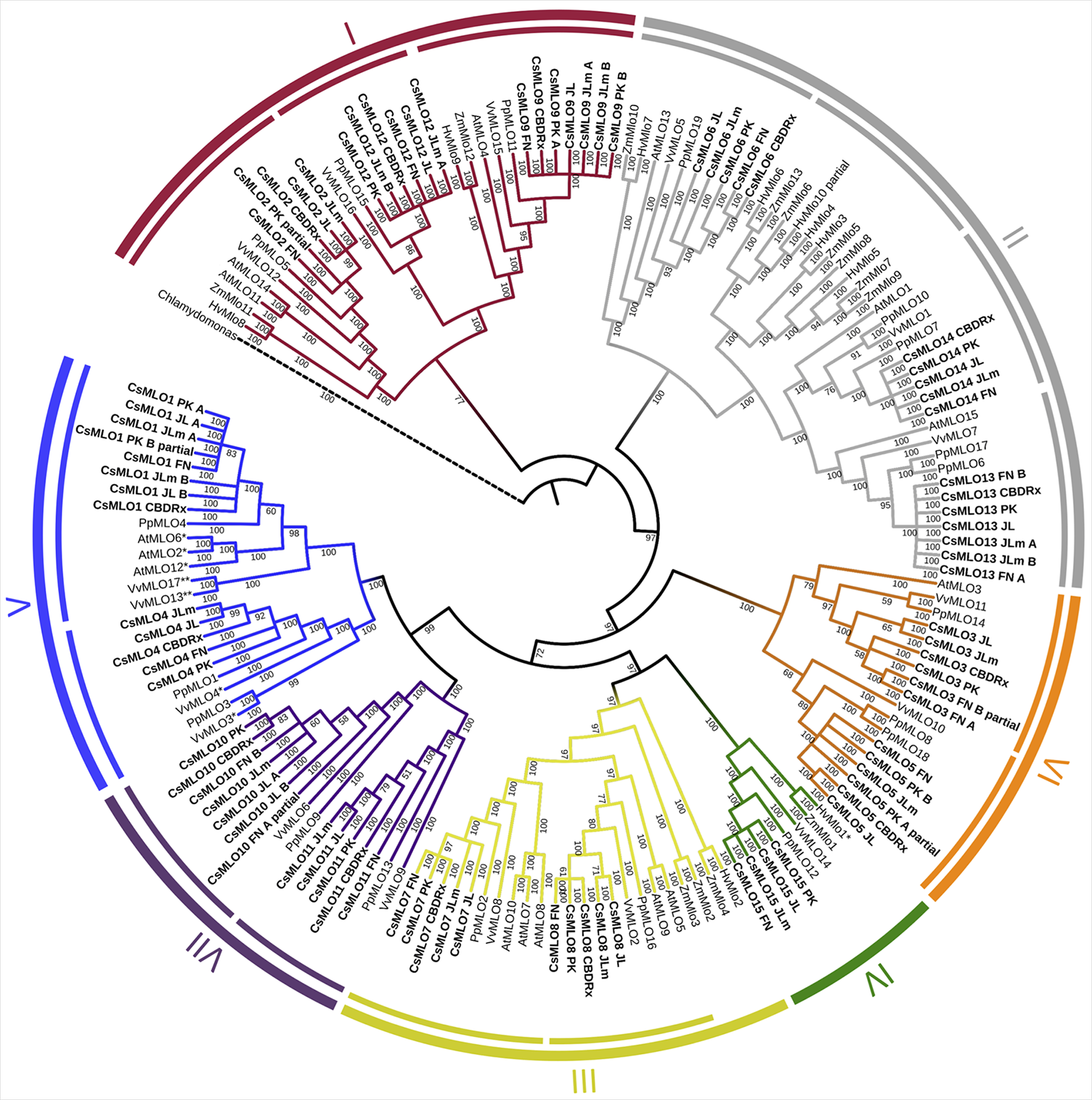

Supplement: Supplementary Figure 1 — Phylogenetic relationships of CsMLOs based on Bayesian inference analysis. Phylogenetic tree of manually curated CsMLO proteins (bold) with MLO proteins from selected species (Arabidopsis thaliana, Prunus persica, Vitis vinifera, Hordeum vulgare, and Zea mays). Chlamydomonas reinhardtii was used as an outgroup. Phylogenetic relationships were estimated using the MrBayes tool implemented on NGPhylogeny.fr, using default parameters. The seven defined clades are indicated, as well as potential subclades identified in this study (inner circles). Number on a node indicates the posterior probabilities of major clades and subclades. MLOs with one asterisk (∗) have been experimentally demonstrated to be required for PM susceptibility (Büschges et al., 1997; Feechan et al., 2008; Wan et al., 2020), while MLOs with two asterisks (∗∗) have been identified as main probable candidates for PM susceptibility (Pessina et al., 2016). [file Image_1.TIFF]

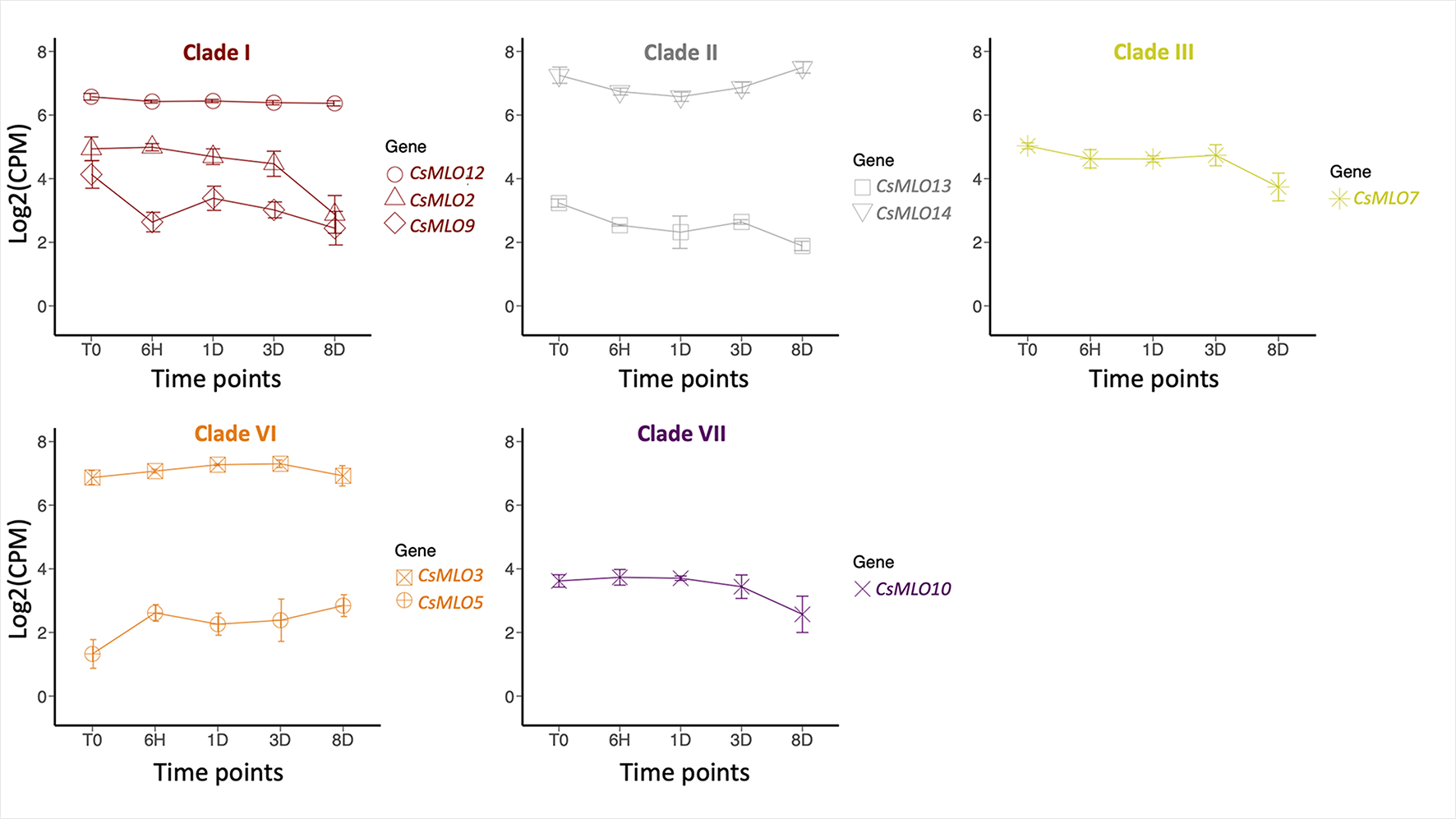

Supplement: Supplementary Figure 2 — Transcriptomic response of CsMLO genes from all clades, except from clade V, following inoculations with powdery mildew. CsMLO genes from clades I, II, III, VI, and VII are displayed in clade-specific plots with different colors depicting the different clades: red (clade I), gray (clade II), yellow (clade III), orange (clade VI), and purple (clade VII). Gene expression is displayed on the y-axis as the average logarithmic value of the counts per million [log2(CPM)] at each time point (displayed on the x-axis, n = per time point). Time points: no infection/control (T0), 6 h post-inoculation (6H), 24 h post-inoculation (1D), 3 days post-inoculation (3D), and 8 days post-inoculation (8D). Error bars at each time point represent the standard deviation (SD). [file Image_2.TIFF]
